# Genistein and daidzein induce apoptosis of colon cancer cells by inhibiting the accumulation of lipid droplets

**DOI:** 10.29219/fnr.v62.1384

**Published:** 2018-05-11

**Authors:** Yu-Si Liang, Wen-Tao Qi, Weiqun Guo, Chun-Ling Wang, Ze-Bin Hu, Ai-Ke Li

**Affiliations:** 1Cereals & Oils Nutrition Research Group, Academy of State Administration of Grain (ASAG), Beijing, The People’s Republic of China; 2Key Laboratory of Food Safety and Sanitation, Ministry of Education, College of Food Engineering and Biotechnology, Tianjin University of Science and Technology, Tianjin, The People’s Republic of China; 3Institute for In Vitro Diagnostic Reagents Control, The National Institutes for Food and Drug Control (NIFDC), Beijing, The People’s Republic of China

**Keywords:** genistein, daidzein, lipid droplets, apoptosis, colon cancer

## Abstract

**Aim:**

The purpose of this study was to investigate the possible mechanisms of genistein (GEN) and daidzein (DAI) in inducing apoptosis of colon cancer cells by inhibition of lipid droplets (LDs) accumulation.

**Methods:**

HT-29 cells were used and treated by GEN or DAI in this paper. LDs accumulation was induced and inhibited by oleic acid (OA) and C75, respectively. The expression changes of LDs-related markers were confirmed by semiquantitative real time-PCR (RT–PCR), Western blotting, and immunofluorescence staining.

**Results:**

GEN and DAI effectively reduced the LDs accumulation and downregulated the expression of Perilipin-1, ADRP and Tip-47 family proteins and vimentin levels. GEN and DAI significantly induced the mRNA expression of PPAR-γ, Fas, FABP, glycerol-3-phosphate acyltransferase (GPAT3), and microsomal TG transfer protein (MTTP), and reduced the mRNA expression of UCP2. Furthermore, the results showed a decrease of PI3K expression by GEN and DAI when compared with OA treatment, and both GEN and DAI can increase the expression of FOXO3a and caspase-8 significantly when these proteins were decreased by OA treatment. GEN is more effective than DAI in inducing cell apoptosis.

**Conclusion:**

Our results demonstrated that GEN and DAI inhibit the accumulation of LDs by regulating LDs-related factors and lead to a final apoptosis of colon cancer cells. These results may provide important new insights into the possible molecular mechanisms of isoflavones in anti-obesity and anti-tumor functions.

Colon carcinoma is one of the leading causes of cancer mortality worldwide (1). Increased risk factors such as age, consumption of high-fat diet, lack of physical activities, alcohol, smoking, environmental pollution, and hereditary are recognized as the cause of the colon cancer (1, 2). Most of the colon cancers are developed by an accumulation of several mutations throughout the lifetime (3). Furthermore, some incident of colon cancers can be lowered or inhibited by more reasonable life style including healthy diet. Epidemiological studies have suggested that a higher dietary intake of soybean products contributes to a lower incidence of colorectal cancer in Asian countries (1, 2).

Soya beans are an abundant source of bioactive compounds of which isoflavonoids have received significant attention due to their potential anticarcinogenic and antiproliferative effects and possible role in many signal transduction pathways (4, 5). There are two major kinds of isoflavones in soybeans: glycosides, including daidzin and genistin;and aglycons, including DAI and GEN (6). Dietary aglycons are known to be absorbed faster and metabolized more effectively than glucosides (7). Another study showed that aglycons exerted more beneficial effects on bone metabolism, with positive impacts on human health, than did glucosides (8). Indeed, numerous studies have shown the prominent component of aglycons, GEN, has anticancer properties (9, 10). Possible mechanisms mainly include an inhibitory effect on tyrosine kinases and DNA topoisomerases, induction of apoptosis, and modulation of the pathway of signal conduction such as PI3K/Akt and Wnt/β-catenin (11). However, the roles of the anticancer effect of aglycons and their molecular targets remain to be further elucidated.

In general, the ability to overcome metabolic stress is a crucial step for cancer cell survival and metastasis (11). It is believed that the accumulation of lipid droplets (LDs) offered the required energy that drives cell proliferation during the process and has been considered a hallmark of tumor progression (12). LDs are organelles constitutively present in adipocytes and serve as lipid stores that provide energy for a number of diverse functions in organisms (13–15). Human colon adenocarcinoma cell lines and colon cancer biopsies from patients have been shown to exhibit an increase in adipose differentiation-related protein (ADRP), which is a major structural protein associated with LD (16). Furthermore, inhibition of LD formation has been proved to be correlated with diminished cancer cell proliferation *in vitro* (17), while the mechanisms involved in this adaptation are still poorly understood (3, 18).

In this study, we show the evidence that the regulation of GEN and DAI induces apoptosis of colon cancer cells by inhibiting the accumulation of lipid droplets.

## Materials and methods

### Cell culture

Human colonic cancer HT-29 cells were purchased from the Institute of Biochemistry and Cell Biology, Shanghai Institutes for Biological Sciences, Chinese Academy of Sciences (Shanghai, China), which was originally offered by the American Type Culture Collection (ATCC), Manassas, VA, USA. The cells were grown in Dulbecco’s Modified Eagle Medium (Gibco) supplemented with 10% fetal bovine serum (Gibco). The cells were cultured at 37°C in a humidified atmosphere of 95% air to 5% CO_2_.

### Cell *t*reatment

Monolayers of human HT-29 cells were treated with genistein (GEN) and daidzein (DAI purchased from Solarbio (Beijing, China) for 48 h at concentrations of 25, 50, 100, 200, and 400 μM, respectively. LDs accumulation was induced with 1 mg/mL oleic acid (OA) (Sigma, St. Louis, MO, USA). During the treatment, cells were placed in serum-free and antibiotic-free medium.

### Cell proliferation

An inhibitory effect of GEN and DAI on proliferation of colon cancer cell lines was evaluated by the MTT [3-(4,5-dimethylthiazol-2-yl)-2,5-diphenyl tetrazolium] assay. HT-29 cells were plated in 96-well plates (5,000 cells per well). After incubation for 24 h, varied concentrations of GEN and DAI were added into each well, and each concentration was repeated in five wells. After 48 h incubation, the medium was aspirated, and 0.5 μg/mL MTT was added. Cells were incubated at 37°C for another 4 h, and the formed formazan product was solubilized with dimethylsulfoxide (DMSO). The optical density (OD) of each well was then measured on an enzyme-linked immunosorbent assay (ELISA) microplate reader (Multiskan EX, Labsystems, Helsinki, Finland) at 570 nm. The effects of GEN and DAI on cell proliferation were assessed as percentage cell viability compared to control cells, which were arbitrarily assigned 100% viability. Each test was performed in triplicate experiments.

### Inhibitors

C75, a fatty acid synthase inhibitor purchased from Sigma-Aldrich Chemical (St. Louis, MO), was used to assess the effect of LDs on protein regulation at a concentration of 50 μg/mL.

### Scanning electron microscopy analysis

The cell suspension (1×10^5^/mL) was seeded on cover slips which were partitioned previously into the wells of 6-well plates. After 24 h, HT-29 cells were treated with 200 μM GEN and DAI, respectively, for 24 and 48 h, followed by pre-fixed glutaraldehyde (2.5%) at 4°C for 1 h. Subsequently, the cells were rinsed thoroughly with phosphate buffered saline (PBS) and post-fixed in OSO_4_ (1%) at 4°C for 1 h; after gold spraying, scanning electron microscopy was applied to observe the change of cell surface morphology.

### Flow cytometry analysis

HT-29 cells were seeded in a 6-well plate and treated with 200 μM GEN and DAI, respectively, for 24 and 48 h, after which they were collected. Subsequently, the cells were washed with cold PBS and fixed by ethanol (70%, v/v). The cells were dissolved in PBS (containing PI, RNase, EDTA, and Triton X-100, pH 7.4) and incubated at 37°C for 30 min, followed by incubation at 4°C for 1 h in the dark. Ulternately, the samples were detected with a flow cytometry (Becton, Dickinson, San Antonio, TX, USA).

### Oil *r*ed-O staining

Monolayers were rinsed three times with PBS, fixed in buffered formalin solution (Sigma) and then stained with Oil Red-O solution (Solarbio, Beijing, China). After rinsing in distilled water, the monolayers were counterstained with Gill’s III and mounted with Prolong Gold antifade reagent (Invitrogen, Carlsbad, CA, USA).

### Boron-dipyrromethene (BODIPY) staining

Monolayers were fixed in formalin and incubated with 1 μg/mL BODIPY (ThermoFisher). Slides were washed, mounted with Prolong Gold antifade reagent (Invitrogen, Carlsbad, CA, USA), and observed under fluorescent microscope (Olympus).

### Immunofluorescence imaging of *v*imentin

Briefly, the cell suspension (1×10^5^/mL) was inoculated on cover slips which were partitioned previously into the wells of a 6-well plate. After 24 h, HT-29 cells were treated with 200 μM GEN and DAI, respectively, for 48 h. Cells were fixed with 3% formaldehyde in PBS (pH 7.4) for 20 min and washed thrice with PBS. Washed cells were permeabilized using 0.2% Triton X-100 and blocked in 2% BSA (B.M.) in PBS. Then the cells were washed thrice with PBS and incubated with the antibody vimentin (dilution 1:200) with 2% BSA in PBS at 37°C for 1 h. The resulting cells were washed thrice with PBS and incubated with fluorescein isothiocyanate- labeled polyclonal goat anti-mouse IgG antibody (dilution 1:200) at 37°C for 1 h. Cells were stained with propidium iodide (4’,6-diamidino-2-phenylindole – DAPI) (Sigma) and scanned by laser scanning confocal microscope. All images were acquired using the same intensity and photodetector gain.

### DAPI staining

The levels of nuclear condensation and fragmentation were observed by means of nucleic acid staining with DAPI (Solarbio, Beijing, China). Briefly, HT-29 cells were plated in 6-well plates (10^5^ cells per well). After treatment, the cells were washed twice with PBS and fixed with methanol (MeOH) and acetic acid (HAc) (3:1, v/v) for 10 min at 4°C. The cells were stained with DAPI (10 mg/mL) for 20 min in the dark and then observed under a fluorescence microscope (Olympus BX41, Japan) within 15 min.

### Protein extraction and *i*mmunoblot

Experimental monolayers were washed with serum-free media, and then total and fractionated proteins were extracted by cell lysis buffer (Cell Signaling Technology, Danvers, MA). The lysates were centrifuged at 12,000 g for 20 min at 4°C. An equal amount of protein after concentration was determined by the Bradford assay (Bio-Rad, Hercules, CA) was loaded on SDS-PAGE and transferred to nitrocellulose membranes (Bio-Rad). After blocking, specific antibodies such as ADRP, FOXO3a, caspase-8, and β-actin from ABclonal Biotechnology (Wuhan, China); Perilipin-1 from Cell Signaling Technology (Danvers, MA); and vimentin from Abcam (Cambridge, MA) were used to perform detection. Finally, each protein was detected using an enhanced chemiluminescence system (GE Healthcare, Chicago, IL, USA). Blot images were digitized (Chemidoc, Bio-Rad, Milan, Italy), and the area of each band was quantified using the computerized imaging system (QuantityOne, Bio-Rad). Relative OD (arbitrary units) was normalized for control bands in each series and for protein loading (as probed by anti-actin blots). Each test was performed in triplicate experiments.

### RNA extraction and semiquantitative RT-PCR procedure

Total RNA was extracted from cultured HT-29 cells using Trizol reagent according to the manufacture’s instruction (TransGen Biotech). RNA concentration was quantified by UV spectrophotometer at 260 and 280 nm. Total RNA was created with first-stand cDNA synthesis kit (TransGen Biotech). Each amplification was performed for 35 cycles, where one cycle profile consisted of denaturation at 94°C for 30 s, annealing at 55°C for 30 s and extension at 72°C for 120 s. The semiquantitative RT-PCR kit was used to detect mRNA levels of PPAR-γ, Fas, UCP2, L-FABP, glycerol-3-phosphate acyltransferase (GPAT3), microsomal TG transfer protein (MTTP), and GAPDH according to the manufacturer’s instructions. The primer sequences that used for semiquantitative RT-PCR are shown in Table 1.

**Table 1:** Primer sequences used for semiquantitative RT-PCR

Gene	Forward	Reverse	Products (bp)
*PPAR-γ*	CCTCACAGCTGTTTGCCAAG	GTGTATCAGCAGTTCCACTCAC	398
*Fas*	ATTGCTTGTGTTTGTGGGTGC	AGCAATCTCTTTGCTCAGGCT	315
*UCP2*	CTTCTGCACCACTGTCATCG	TCACACTTGGCTGCTACTCAC	275
*L-FABP*	AGCGGCTCACATACCCTAAT	TTATGAAGCACCCTGGGCAT	478
*GPAT3*	CACTTTTTAGAGCCAAAAGGAATCT	AAGTCAGCAGACAAGGGTGG	432
*MTTP*	AGCACATCCAGGTCACCTCT	CCTGAACGCTGCATCTTACC	337
*GAPDH*	GGACTCATGGTATGAGAGCTGG	GATGGCATGGACTGTGGTCT	227

### Statistical analysis

The experiments were repeated three times, and the mean values were analyzed by a two-tailed unpaired *t*-test. The results were expressed as mean ± SD. The level of *p* < 0.05 was considered to be statistically significant.

## Results

### Genistein and daidzein inhibit the proliferation of HT-29 cells

**Fig. 1:**
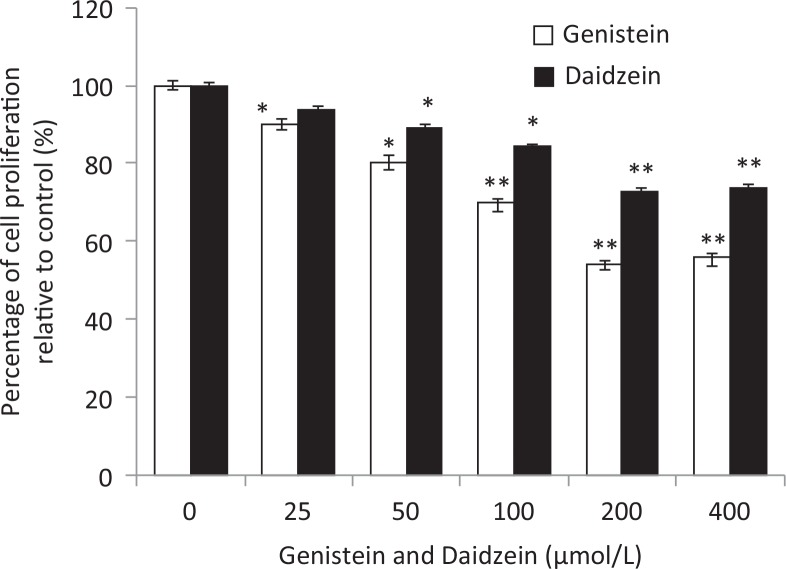
The effect of GEN and DAI on proliferation of HT-29 cells. HT-29 cells were exposed to GEN and DAI for 48 h. Proliferation was determined by MTT assessed as percentage cell viability compared to control cells, which were arbitrarily assigned 100% viability. The results were expressed as means ± SD (*n* = 6). *p < 0.05, **p < 0.01 compared with control cells.

The treatment of HT-29 cells by GEN and DAI at 0, 25, 50, 100, 200, and 400 μM for 48 h is illustrated in Fig. 1. The results showed that both GEN and DAI inhibited the growth of HT-29 cells remarkably in a dose-dependent manner. The inhibition of proliferation could be up to 46 and 34% at the concentration of 200 μM for GEN and DAI, respectively. Above 200 μM, no alterations of cell proliferation inhibition were observed with the increase of both GEN and DAI concentrations. Thus, all the treatments of cells in the following experiment were carried out with 200 μM GEN or DAI for 48 h.

### Genistein and daidzein induce apoptosis of HT-29 cells

**Fig. 2:**
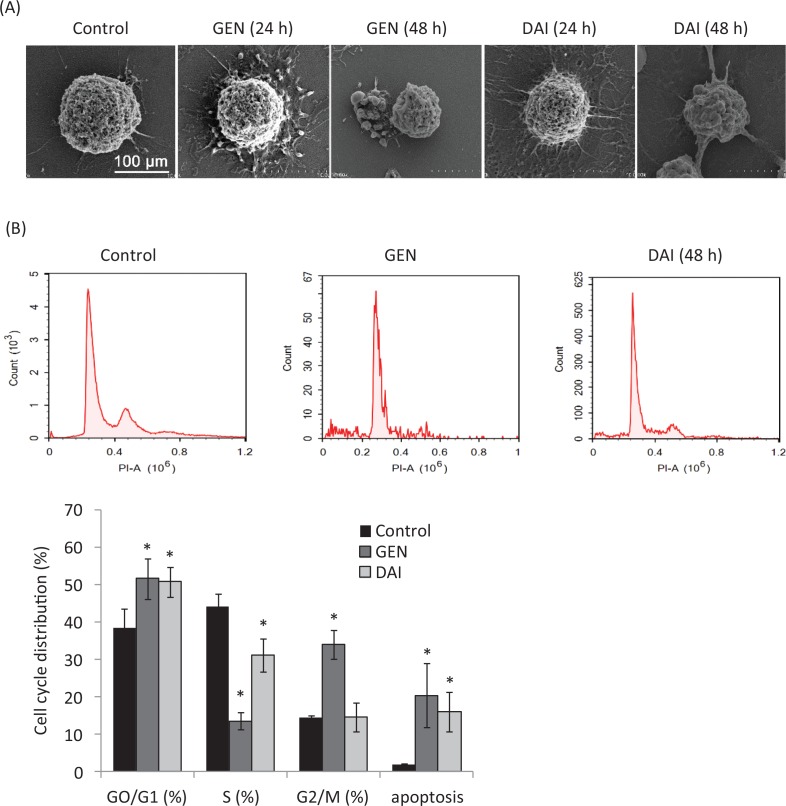
The apoptosis of HT-29 cells induced by GEN and DAI. (A) Scanning electron micrographs of HT-29 cells after treatment with GEN (200 μM) and DAI (200 μM), respectively, for 24 and 48 h. Bar = 100 μm. (B) Cell cycle distribution and apoptosis rate of HT-29 cells by flow cytometry after treatment with GEN (200 μM) and DAI (200 μM), respectively for 48 h. The results were expressed as means ± SD (*n* = 3). *p < 0.05 compared with control cells.

Cell apoptosis is a procedure of cell disintegration accompanied by featured morphological variation such as condensation of chromatin, loss of microvilli, blebbing, and apoptotic body formation (19). The morphology changes of HT-29 cells treated with GEN or DAI were observed by a scanning electron microscopy in this article. The results showed that cells with normal volumes and rich microvilli of the control group were kept intact and had integral cell membranes (Fig. 2a), whereas cells treated with GEN or DAI showed typical pre-apoptotic morphologies, characterized by loss of microvilli, cell shrinkage, and blebbing formation at 24 h, and loss of microvilli and appearance of apoptotic bodies were observed significantly at 48h (Fig. 2a). The cell-cycle phase distribution and the ratios of apoptotic cells were further determined by flow cytometry with PI staining. The percentage of cells in G1, S, and G2/M phase was calculated using multi-cycle software. The results showed a significant increase of cells in the G0/G1 phase from 38.48 ± 5.10% for control to 51.69 ± 5.45% and 50.76 ± 4.07% (*p* < 0.05) for GEN and DAI treatments, respectively, and the apoptotic rate increased significantly (*p* < 0.05) from 2.01 ± 0.15% for control to 20.49 ± 8.46% and 16.11 ± 5.23% for GEN and DAI, respectively (Fig. 2b). These results confirmed that both GEN and DAI could inhibit the proliferation of HT-29 cells by induction of apoptosis.

### Genistein and daidzein reduced the influences of OA on HT-29 cells

**Fig. 3:**
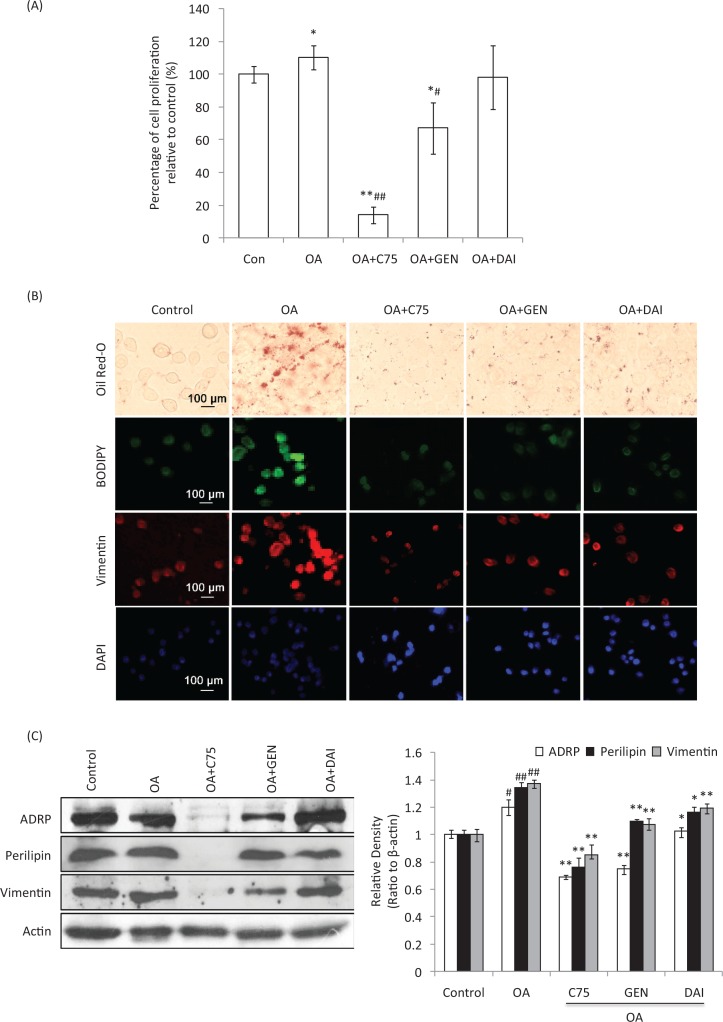
The effect of GEN and DAI on the proliferation, accumulation of lipid droplets, and the expression of lipid droplets (LDs)-related proteins in HT-29 cells. LDs accumulation was induced with 1 mg/mL oleic acid (OA) (Sigma). Cells were treated by GEN (200 μM) and DAI (200 μM), respectively, for 48 h. Fatty acid synthase inhibitor C75 was used to assess the effect of LDs on protein regulation at a concentration of 50 μg/mL. (A) Proliferation was determined by MTT assessed as percentage cell viability compared to control cells, which were arbitrarily assigned 100% viability. The results were expressed as means ± SD (*n* = 6). #*p* < 0.05, ##*p* < 0.01 compared with control cells; *p < 0.05, **p < 0.01 compared with OA-treated cells. (B) LDs density was determined by Oil Red-O and BODIPY staining. The expressions of cytoskeletal proteins, vimentin, were examined by immunofluorescence staining. Morphological evidence of cell apoptosis was measured by DAPI staining. The stained cells were observed under a laser confocal fluorescence microscope (×400). Bar = 100 μm. (C) The expressions of ADRP, perilipin, and vimentin were analyzed by western blot. Density of the bands was quantified by densitometry analysis and was presented after normalization by β-actin. The results were expressed as means ± SD (*n* = 3). ^#^ *p* < 0.05, ^##^ *p* < 0.01 compared with control cells; *p < 0.05, **p < 0.01 compared with OA-treated cells.

To determine the effects of GEN and DAI on OA-treated cells, HT-29 cells were loaded with LDs using 1 mg/mL OA,  while the fatty acid synthase inhibitor C75 was used to inhibit the OA-induced LDs accumulation. MTT results showed that OA can induce the proliferation of HT-29 cells 10% significantly at 48 h compared to control cells (*p* < 0.05). C75 significantly decreased the proliferation compared to both control and OA-treated cells (*p* < 0.01). The addition of GEN was also found to inhibit the proliferation induced by OA, while the inhibition of DAI was not significant (*p* > 0.05) (Fig. 3a). Oil Red-O staining showed that the density of LDs was remarkably increased by OA at 48 h compared to control cells. The addition of C75 significantly decreased the LDs aggregation during OA treatment. The additions of GEN or DAI were also found at different levels of decrease of LDs accumulation induced by OA similar to the presence of C75 (Fig. 3b).This was confirmed by immunofluorescent staining of neutral lipids (BODIPY) and vimentin (Fig. 3b). Perilipin-1, ADRP and Tip-47 (PAT) proteins, ADRP and perilipin-1, and vimentin were further determined by using Western blotting. It was found that all these proteins were induced significantly by OA addition (*p* < 0.05). While both GEN and DAI can significantly reduce the increased expression of ADRP by 14.6 and 37.5%, respectively, compared with OA-treated cells (*p* < 0.01), the reduction of perilipin-1 was 14.6 and 13.1%, respectively (*p* < 0.05) (Fig. 3c). Similar results were found when vimentin expression was analyzed, and the percentage of reduction by GEN and DAI was 21.8 and 13.1%, respectively, compared with OA-treated cells (*p* < 0.05) (Fig. 3c). These results indicate that GEN or DAI can reduce the OA-induced proliferation and effectively reduce the LDs accumulation by downregulating PAT family proteins and vimentin levels in HT-29 cells.

### Effects of genistein and daidzein on the mRNA expression of LD-associated genes in HT-29 cells

**Fig. 4:**
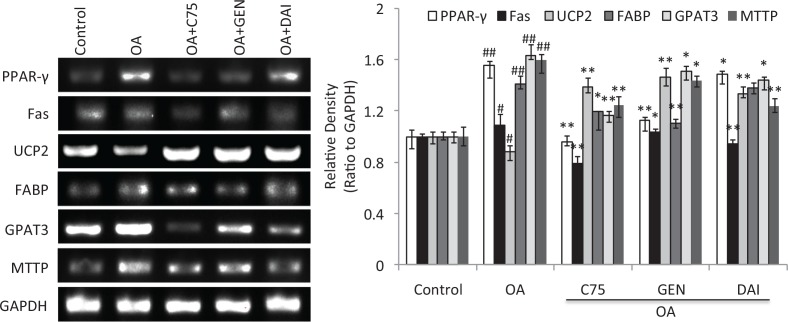
The effect of GEN and DAI on the mRNA expressions of LD-associated genes in HT-29 cells. mRNA expressions of *PPAR-γ*, *Fas*, *FABP*, *GPAT3*, *MTTP*, and *UCP2* were determined by RT-PCR analysis. Quantifications of the mRNA were normalized by *GAPDH*. HT-29 cells were treated with GEN (200 μM) and DAI (200 μM), respectively, for 48 h. The results were expressed as means ± SD (*n*= 3). ^#^ *p* < 0.05, ^##^ *p* < 0.01 compared with control cells; *p < 0.05, **p < 0.01 compared with OA-treated cells.

In addition to the changes of LDs accumulation and its related proteins, the mRNA expressions of LD-associated genes in the cells were further evaluated using semiquantitative RT-PCR assay. The results showed that OA can significantly induce the mRNA expression of PPAR-γ, Fas, FABP, GPAT3, and MTTP and reduce the mRNA expression of UCP2 (*p* < 0.05) (Fig. 4). While both GEN and DAI as well as C75 significantly reversed the change of decreased mRNA expression induced by OA (*p* < 0.05) (Fig. 4), these data demonstrate that both GEN and DAI can decrease the LDs density by regulation of the mRNA expression of LD-associated genes in HT-29 cells.

### Effects of genistein and daidzein on the expression of PI3K, FOXO3a*,* and Caspase-8 proteins

**Fig. 5:**
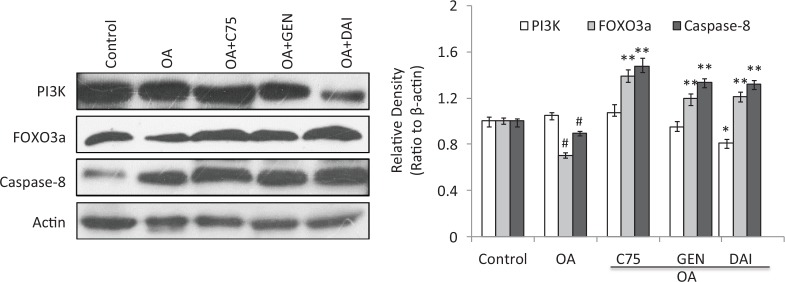
The effect of GEN and DAI on the expressions of PI3K, FOXO3, and caspase-8. Western blot analysis was carried out to demonstrate the expression of proteins. Densities of the bands were quantified by densitometry analysis. Data are presented after normalization by β-actin. HT-29 cells were treated by GEN (200 μM) and DAI (200 μM), respectively, for 48 h. The results were expressed as means ± SD (*n*= 3). ^#^ *p* < 0.05, ^##^ *p* < 0.01 compared with control cells; *p < 0.05, **p < 0.01 compared with OA-treated cells.

The results showed a decrease of PI3K expression by GEN and DAI when compared with OA treatment, although the reduction by C75 and GEN was not remarkable. Both GEN and DAI can increase the expression of FOXO3a and caspase-8 significantly *(p* < 0.05) as well as C75 when these proteins were decreased by OA treatment (Fig. 5). Similar results as GEN and DAI were found when the C75 was added to the HT-29 cells. These results demonstrated that the expressions of FOXO3a and caspase-8 are negatively associated with LDs accumulation, and all can be upregulated by GEN and DAI in HT-29 cells.

## Discussion

**Fig. 6:**
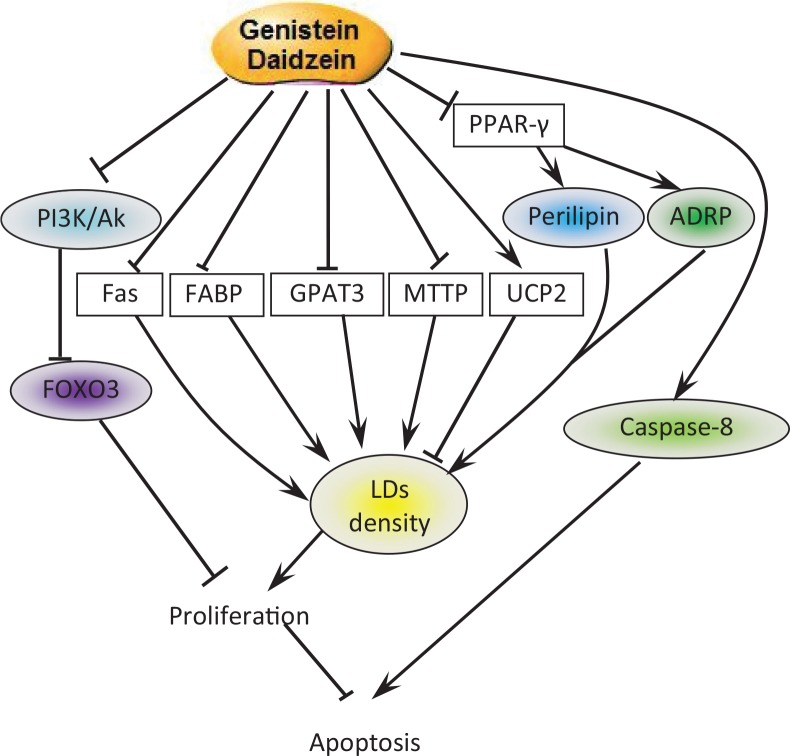
Pathways involved in cell apoptosis and LDs decrease induced by GEN and DAI. GEN and DAI inhibit the accumulation of LDs by downregulating the expression of LDs-related proteins perilipin and ADRP combined with the regulations of lipid metabolism-associated genes. On the other hand, GEN and DAI induce caspase-8 expression and inhibit PI3K activity, which results in an increased level of FOXO3. All these functions lead to a final apoptosis of colon cancer cells.

Isoflavones, especially GEN, has been proved to have anticancer properties by many researches (20). This study revealed a novel mechanism that GEN and DAI utilize to inhibit proliferation. GEN and DAI inhibit proliferation and induce apoptosis of colon cancer cells by inhibiting the accumulation of lipid droplets. Upstream, GEN and DAI induced FOXO3 and caspase-8 activity by targeting PI3k pathway. Downstream, GEN and DAI inhibit OA-induced LDs accumulation by mediated PAT and vimentin proteins via PPAR-γ pathway accompanied by the regulation of the mRNA expression of LD-associated genes (Fig. 6).

In the present study, we demonstrated that the exposure of HT-29 cells to GEN and DAI results in a dose-dependent inhibition of cell proliferation and strongly promotes apoptosis. GEN is a more effective inhibitor of cell proliferation than DAI. These results are consistent with several previous studies (21, 22).

Several studies have shown increased LD density within cancer cells (12, 23), yet the relationship between LDs and the cell cycle is unclear. LDs were previously viewed as storage depots by which colon cancer cells support their high fuel and energy demands during tumor progression (23, 24). In the present study, GEN and DAI were found to reduce LDs density and the cell proliferation induced by OA. Furthermore, GEN and DAI were found to decrease the PAT protein family ADRP and perilipin-1. ADRP is a kind of adipocyte factor and an essential protein of lipid metabolism that expresses in the early stage of adipocyte differentiation; it plays an important role in promoting the formation of lipid droplets (16). Perilipin-1 is a highly phosphorylated adipocyte protein that locates at the surface of the lipid droplet and serves important functions in double-regulating triacylglycerol metabolism (25). Upregulation of LD-associated proteins such as PLIN family, CIDEC, CIDEA, HILPDA, FITM1, FITM2, and G0S2 by PPARs including PPAR-γ has been considered a mechanism to link uptake of lipids to regulation of lipid storage capacity (26). In this article, we found a significant decrease of PPAR-γ mRNA expression by GEN and DAI, especially GEN. These results demonstrate that the inhibitory effects of GEN and DAI on LDs accumulation are mediated through PPAR-γ pathway.

It has been confirmed that the fatty acid synthase (Fas) is an essential component and plays an important role in fatty acid biosynthesis metabolism (27). Increased expression of Fas has been proved to provide a survival advantage to colorectal cancer cells via upregulation of cellular respiration (28). Liver fatty acid binding protein (L-FABP) is an abundant cytosolic fatty acid carrier involved in the long chain FA transport, which can be active by PPAR-γ (29). GPAT3 is the rate-limiting enzyme involved in triglycerides (TGs) synthesis, and its overexpression in mammalian cells results in increased TG formation (30). MTTP is a protein required for lipoprotein assembly and secretion, whose gene was found to be strongly associated with fatty acid profile and positively mediated the fat deposits (31, 32). All these LDs-promoted factors were found can to be significantly reduced by GEN and DAI. Besides, it is interesting to note that both GEN and DAI significantly increased mRNA levels of the thermogenesis-related protein UCP2 that can improve abnormal lipid metabolism by suppressing lipogenesis and promoting fatty acid (33). The relationship between vimentin and LDs was already described (34). And the specific binding of perilipin-1 and vimentin were found to protect LDs and against lipase activity in human adipose cells (35). In this article, we found that the level of vimentin promoted by OA can be significantly decreased by the addition of GEN and DAI. All these results strongly demonstrate that GEN and DAI have a dual function in regulating both cell proliferation and LD-mediated metabolic needs for sustained cell growth.

The effects of GEN and DAI on PI3K, FOXO3, and caspase-8 expression were further determined. It has been previously demonstrated by several reports that GEN inhibits proliferation in colon cancer cells via PI3K/Akt, a pathway known to be critical to colon cancer progression (36–38). We previously found that GEN-inhibited PI3K/Akt activation leads to inactivation of FOXO3, which negatively regulates proliferation in colon cancer cells (36). Here we confirmed both GEN and DAI significantly increased the level of FOXO3. Furthermore, we found that GEN and DAI significantly increased the caspase-8 expression that has been known as a key mediator during the executional phase of apoptosis (39). The present study showed that GEN and DAI treatment caused activation of caspase-8 whose activity was inhibited by OA, suggesting that GEN and DAI induce cell death through a caspase-dependent mechanism.

In summary, our results demonstrated that GEN and DAI inhibit the accumulation of LDs by downregulating the expression of LDs-related proteins, perilipin-1 and ADRP, combined with the regulations of lipid metabolism-associated genes. Moreover, GEN and DAI induce caspase-8 expression and inhibit PI3K activity, which results in an increased level of FOXO3.All these functions lead to a final apoptosis of colon cancer cells. We speculate this possible pathway to demonstrate the GEN and DAI mechanisms of inhibition of LD accumulation and apoptosis in HT-29 cells. These results provide important new insights into the possible molecular mechanisms of isoflavones, in addition to its potential as a novel candidate as an anti-obesity and anti-tumor agent.
